# Supplementary material for: Taming the reference genome jungle: the refget sequence collection standard
Source: bioRxiv. 2025 Oct 6:2025.10.06.680641. Preprint. [Version 1] doi: 10.1101/2025.10.06.680641 (PMC12632599; doi:10.1101/2025.10.06.680641)
Supplement: Supplement 5 [file NIHPP2025.10.06.680641v1-supplement-5.pdf]

bioRxiv preprint doi: https://doi.org/10.1101/2025.10.06.680641; this version posted October 6, 2025. The copyright holder for this preprint (which was not certified by peer review) is the author/funder, who has granted bioRxiv a license to display the preprint in perpetuity. It is made available under aCC-BY 4.0 International license.

Supplemental Figures

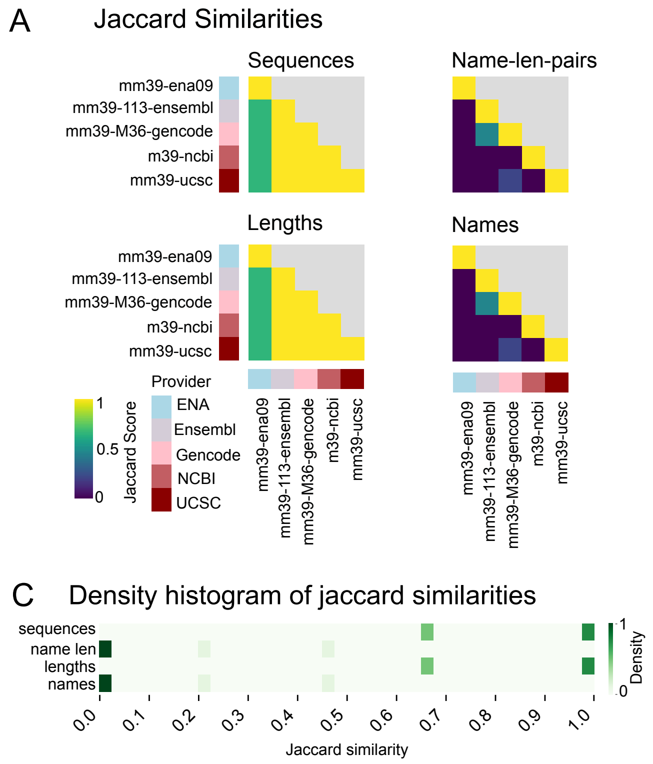

Supplemental Figure 1: Results of comparison of primary mouse assemblies across 5 major providers. *A) Jaccard similarity scores for sequences, name-length-pairs, lengths, and names. B) OPA and OPB scores for sequences, name-length-pairs, lengths, and names. C) Density map of Jaccard similarity scores for 10 pairwise comparisons of the 5 major providers.*

bioRxiv preprint doi: https://doi.org/10.1101/2025.10.06.680641; this version posted October 6, 2025. The copyright holder for this preprint (which was not certified by peer review) is the author/funder, who has granted bioRxiv a license to display the preprint in perpetuity. It is made available under aCC-BY 4.0 International license.

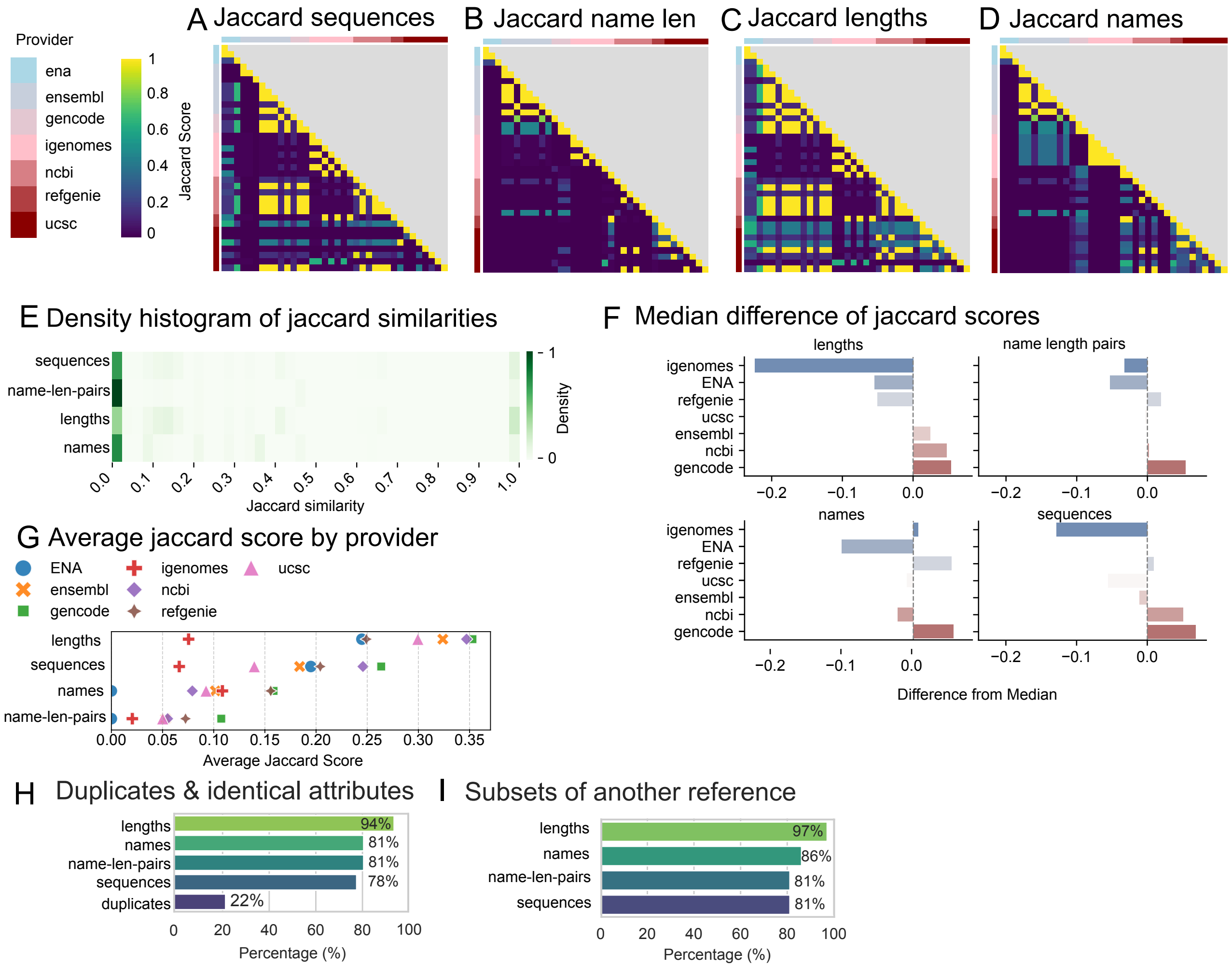

Supplemental Figure 2: Comparing Jaccard similarities for 36 mouse reference genomes, grouped by provider. *A) Jaccard similarity scores for sequences. B) Jaccard similarity scores for name length pairs C) Jaccard similarity scores for lengths. D) Jaccard similarity scores for names. E) Density map of Jaccard similarity scores for 630 pairwise comparisons. F) Median difference plots for each attribute grouped by provider G) Average jaccard scores for each provider H) Bar chart showing percentages of duplicates and identical references based on attributes. I) Bar chart showing percentage of references that are subsets of each other based on attributes.*

bioRxiv preprint doi: https://doi.org/10.1101/2025.10.06.680641; this version posted October 6, 2025. The copyright holder for this preprint (which was not certified by peer review) is the author/funder, who has granted bioRxiv a license to display the preprint in perpetuity. It is made available under aCC-BY 4.0 International license.

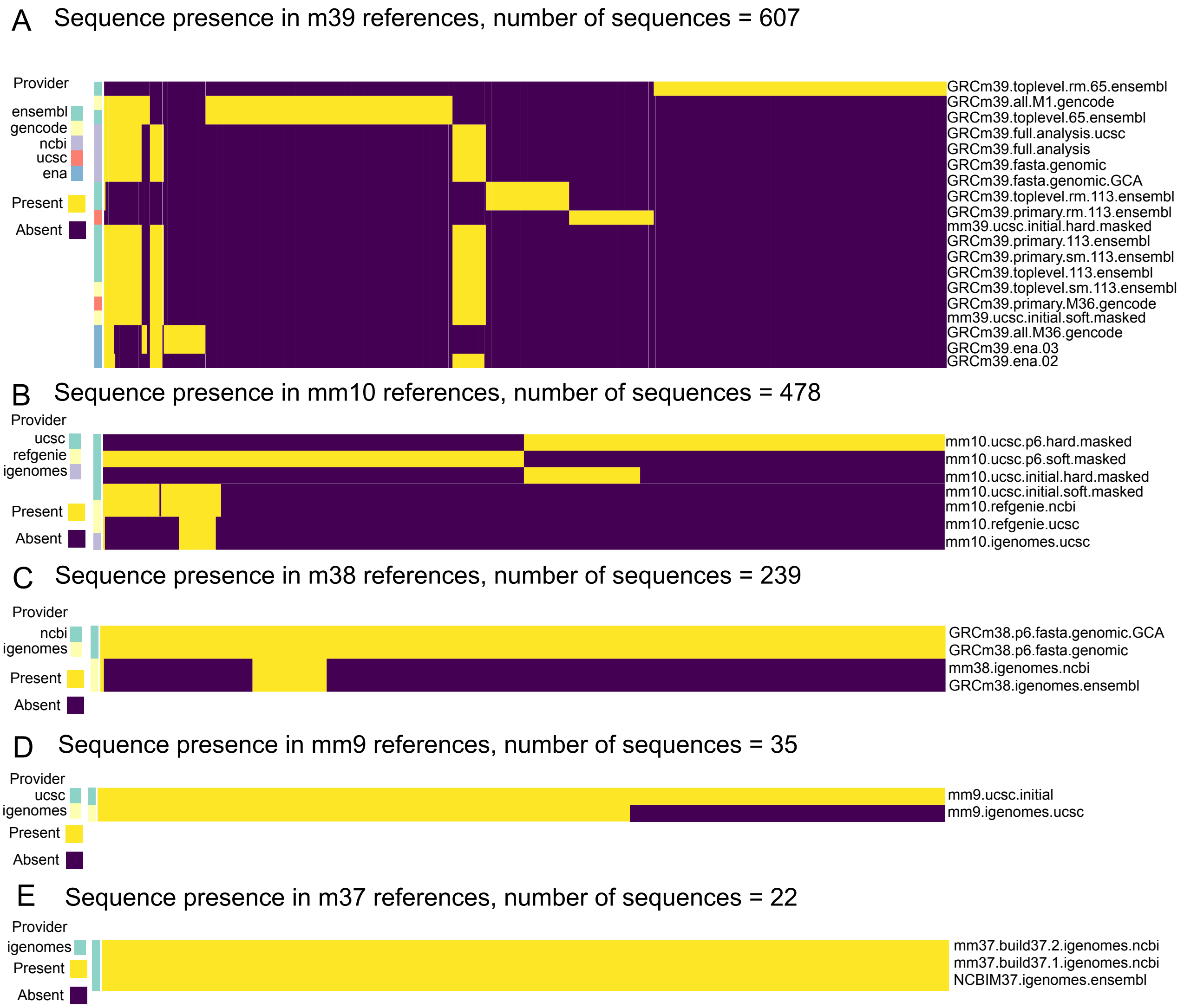

Supplemental Figure 3: Comparing the presence of sequences within mouse references. *A) Comparing 607 sequences within m39 references B) Comparing 478 sequences within mm10 references C) Comparing 239 sequences within m38 references D) Comparing 35 sequences within mm9 references E) Comparing 22 sequences within m37 references.*

bioRxiv preprint doi: https://doi.org/10.1101/2025.10.06.680641; this version posted October 6, 2025. The copyright holder for this preprint (which was not certified by peer review) is the author/funder, who has granted bioRxiv a license to display the preprint in perpetuity. It is made available under aCC-BY 4.0 International license.

Supplemental Tables

Genome	Common	Authority	Description	Top-level digest	N
hg38-toplevel-113-ensembl	hg38	ensembl	top level fasta of GRCh38 release 113	5ryrHdbJHIgyZuE29h5uzITRL4kinZWG	706
GRCh38-p14-47-gencode	hg38	gencode	release 47 of gencode GRCh38	tkRdBlSp5hewK4OpEJC87J9pw-ac0vOa	706
GRCh38.p14-fasta-genomic	hg38	ncbi	GCF_000001405.40_GRCh38.p14_genomic from NCBI	u1HyLgIlq8M_XvEwy0oGqAvKGHJMGtxH	705
hg38-p14-ucsc	hg38	ucsc	hg38 from UCSC patch 14	a_WL8OC7sFJfjux5m11M2bKl0dYepA1x	711
hg38-ddbj	hg38	ddbj	ddbj hg38.fa.gz	NTeQ1GQMt2ocCFkS8Z3_qkvetZjabWSt	455
GRCh38-ena-29	hg38	ENA	ref genome hosted on ENA Genome Reference Consortium Human Build 38 patch release 14 (GRCh38.p14)	lWRRNMNypacEjnJCy-AYiDNUPy1brQGC	68

Supplemental Table S1: 6 major human reference providers. *This table shows major providers used for the core analysis. N=number of sequences present in the reference genome.*

Genome	Common	Authority	Description	Top-level digest	N
GRCm39-fasta-genomic	mm39	ncbi	GCF_000001635.27_GRCm39_genomic from NCBI	fXjBOJjw-DYsSnnfDBl5vtZu1N7lbnUl	61
GRCm39-toplevel-113-ensembl	mm39	ensembl	ensembl 113 top level	XVhfRj6PCzLoGiEeXjpFv7vriVN02aPc	61
mm39-ucsc-initial-soft-masked	mm39	ucsc	mm39 initial ucsc soft masked	KPagVaXI4XwQ1D0L0EW4eEuVl-otaAtX	61
GRCm39-all-M36-gencode	mm39	gencode	gencode all release m36 mm39	WsOG-InFnIta0rqSy1KUBjrFrukbnE5j	61
GRCm39-ena-09	mm39	ENA	ref genome hosted on ENA Genome Reference Consortium Mouse Build 39	ABEupc6KHmxtHGarfWFXTmu9mUcNnfoM	40

Supplemental Table S2: 5 major mouse reference providers. *This table shows major providers used for the core analysis. N=number of sequences present in the reference genome.*

Genome	Common	Authority	Description	Top-level digest	N
GRCm39-fasta-genomic	mm39	ncbi	GCF_000001635.27_GRCm39_genomic from NCBI	fXjBOJjw-DYsSnnfDBl5vtZu1N7lbnUl	61
GRCm39-toplevel-113-ensembl	mm39	ensembl	ensembl 113 top level	XVhfRj6PCzLoGiEeXjpFv7vriVN02aPc	61
mm39-ucsc-initial-soft-masked	mm39	ucsc	mm39 initial ucsc soft masked	KPagVaXI4XwQ1D0L0EW4eEuVl-otaAtX	61
GRCm39-all-M36-gencode	mm39	gencode	gencode all release m36 mm39	WsOG-InFnIta0rqSy1KUBjrFrukbnE5j	61
GRCm39-ena-09	mm39	ENA	ref genome hosted on ENA Genome Reference Consortium Mouse Build 39	ABEupc6KHmxtHGarfWFXTmu9mUcNnfoM	40

Supplemental Table S3: *Selection of hg19 patch 13 references. N=number of sequences present in the reference genome.*

Genome	Common	Authority	Description	Top-level digest	N
GRCh38.p0-fasta-genomic	hg38	ncbi	GCF_000001405.26_GRCh38_genomic from NCBI	XemD97fxYMS4q-FBm_n5CHQgmzh1_67a	455
GRCh38.p1-fasta-genomic	hg38	ncbi	GCF_000001405.27_GRCh38.p1_genomic from NCBI	P33s5fSkktH60MccIpfLNHAA79fJyxlt	471
GRCh38.p2-fasta-genomic	hg38	ncbi	GCF_000001405.28_GRCh38.p2_genomic from NCBI	t-g5olKSC0DsZHICbi3cYR3jF-7i4xaK	486
GRCh38.p6-fasta-genomic	hg38	ncbi	GCF_000001405.32_GRCh38.p6_genomic from NCBI	9VlpW2Uty7rQNSBbYy-nYCNOHqyYoBTt	521
GRCh38.p7-fasta-genomic	hg38	ncbi	GCF_000001405.33_GRCh38.p7_genomic from NCBI	e38absAQBVXM4If9Mp9UhrPrqNGOklZz	525
GRCh38.p8-fasta-genomic	hg38	ncbi	GCF_000001405.34_GRCh38.p8_genomic from NCBI	41Q6kDYWPOs4h-tpnmW0xyNosJFgX8tH	543
GRCh38.p12-fasta-genomic	hg38	ncbi	GCF_000001405.38_GRCh38.p12_genomic from NCBI	CbeNHqwFVEdeNKe6Obm77fX6U9-fzVUL	594
GRCh38.p13-fasta-genomic	hg38	ncbi	GCF_000001405.39_GRCh38.p13_genomic from NCBI	d2GjQek3_I1_zkq4gLHTUI50BptQsoqZ	639
GRCh38.p14-fasta-genomic	hg38	ncbi	GCF_000001405.40_GRCh38.p14_genomic from NCBI	u1HyLgIlq8M_XvEwy0oGqAvKGHJMGtxH	705

Supplemental Table S4: *Selection of human NCBI GRCh38 patches. N=number of sequences present in the reference genome.*

bioRxiv preprint doi: https://doi.org/10.1101/2025.10.06.680641; this version posted October 6, 2025. The copyright holder for this preprint (which was not certified by peer review) is the author/funder, who has granted bioRxiv a license to display the preprint in perpetuity. It is made available under aCC-BY 4.0 International license.

Genome	Common	Authority	Description	Top-level digest	N
GRCh38.p14-fasta-no-alt-analysis	hg38	ncbi	GCA_000001405.15 GRCh38_no_alt_analysis from NCBI	EiFob05aCWgVU_B_Ae0cypnQut3cxUP1	195
GRCh38.p14-fasta-full-analysis	hg38	ncbi	GCA_000001405.15_GRCh38_full_analysis_set from NCBI	EGlYk1stOsAjmTALWkqKDqtZOSkyA_YH	456
GRCh38.p14-fasta-genomic	hg38	ncbi	GCF_000001405.40_GRCh38.p14_genomic from NCBI	u1HyLgIlq8M_XvEwy0oGqAvKGHJMGtxH	705
GRCh38.p14-fasta-full-analysis-plus-hs38d1	hg38	ncbi	GCA_000001405.15_GRCh38_full_analysis_set from NCBI plus hs38d1 decoy sequences	MkXYxV2-83BEcPmzskEVGJsJ7Qkb--gX	2841
GRCh38.p14-fasta-no-alt-plus-hs38d1	hg38	ncbi	GCA_000001405.15_GRCh38_no_alt_plus_hs38d1_analysis_set from NCBI plus hs38d1 decoy sequences	7hP7E8o-q6H8qcqMHBEIYdbAK49PoEUZ	2580
GRCh37.p13-fasta-no-alt-analysis	hg19	ncbi	GCA_000001405.14_GRCh37.p13_no_alt_analysis from NCBI	DvAlkUMPq7CRnTYAfXGkQmAOfFqVMZHE	85
GRCh37.p13-fasta-full-analysis	hg19	ncbi	GCA_000001405.14_GRCh37.p13_full_analysis_set from NCBI	kgTizBhIBf5BeEMTqBuyzRBi2AlwTRj6	298
GRCh37.p13-fasta-genomic	hg19	ncbi	GCF_000001405.25_GRCh37.p13_genomic from NCBI	XJWKh8nsSqBFfcU0DIHMZohYyCWF-vcA	297
hg38-initial-ucsc	hg38	ucsc	hg38 from UCSC representing initial 2013 release	NTeQ1GQMt2ocCFkS8Z3_qkvetZjabWSt	455
hg38-p14-ucsc	hg38	ucsc	hg38 from UCSC patch 14	a_WL8OC7sFJfjux5m11M2bKl0dYepA1x	711
hg38-p14-masked-ucsc	hg38	ucsc	hg38 masked from UCSC patch 14	xEg2q8K9gV4027DMTCiaUGLCcrqySglR	455
hg38-p14-analysisSet-ucsc	hg38	ucsc	hg38 analysisSet from UCSC patch 14	jFm0Uca8a7vK2cbuIQgBopjBilgCFheD	195
hg19-initial-ucsc	hg19	ucsc	hg19 from UCSC representing initial 2009 release	ThZcNYiLuWWL86NdJ8dvvJG15K9mW3Fo	93
hg19-masked-ucsc	hg19	ucsc	masked hg19 from UCSC representing initial 2009 release	H3RA3Jez6oqMOW87LGuwtSgKQqTgWVxx	93
hg19-p13-full-analysis-ucsc	hg19	ucsc	full analysis set hg19 p13 from ucsc	-qU7PUmse_-pilikFDTJKyrt2_QJvUFy	298
hg19-p13-no-alt-analysis-ucsc	hg19	ucsc	no alt analysis set hg19 p13 from ucsc	svwHqvgassl0loigdqVIQJdjo7NWDIx3	85
hg19-p13-plusMT-ucsc	hg19	ucsc	hg19 p13 plusMT from ucsc	6pxZqxG0TYtyVb8yp14ONpaxZ8msQqKr	298
hg19-p13-plusMT-masked-ucsc	hg19	ucsc	hg19 p13 plusMT masked from ucsc	q5Gn6dl5HbkZe6sRs9CZVGwGd1-XwyAq	298
hg38-primary-113-ensembl	hg38	ensembl	primary fasta of GRCh38 release 113	oLfPx0NOBKKXMIngGeQ4YewtU4Ge_wKz	194
hg38-toplevel-113-ensembl	hg38	ensembl	top level fasta of GRCh38 release 113	5ryrHdbJHIgyZuE29h5uzITRL4kinZWG	706
hg38-alt-113-ensembl	hg38	ensembl	alt sites fasta of GRCh38 release 113	0OgP0NkIM22lVYT5AMmkbb9knKDhk4I6	512
hg38-alt-rm-113-ensembl	hg38	ensembl	alt rm sites fasta of GRCh38 release 113	h_kOcvPobU9it_QR1LjPqeNpM56xjEJQ	512
hg38-toplevel-rm-113-ensembl	hg38	ensembl	top level rm fasta of GRCh38 release 113	F9zeFn6M4EN4KGAJush7rZEU3GROoeNP	706
hg38-primary-rm-113-ensembl	hg38	ensembl	primary fasta rm fasta of GRCh38 release 113	RGSkyOkQ4qnSLhjrR_e3AI51Ac-RohAL	194
hg38-alt-sm-113-ensembl	hg38	ensembl	alt sm sites fasta of GRCh38 release 113	0OgP0NkIM22lVYT5AMmkbb9knKDhk4I6	512
hg38-toplevel-sm-113-ensembl	hg38	ensembl	top level sm fasta of GRCh38 release 113	5ryrHdbJHIgyZuE29h5uzITRL4kinZWG	706
hg38-primary-sm-113-ensembl	hg38	ensembl	primary fasta sm fasta of GRCh38 release 113	oLfPx0NOBKKXMIngGeQ4YewtU4Ge_wKz	194
GRCh38-p14-47-gencode	hg38	gencode	release 47 of gencode GRCh38	tkRdBlSp5hewK4OpEJC87J9pw-ac0vOa	706
GRCh38-primary-assembly-47-gencode	hg38	gencode	release 47 of gencode primary assembly GRCh38	tmnbiAyj2fke68d_TYjq2g487US8C15r	194
GRCh37-primary-assembly-47-gencode	hg19	gencode	release 47 of gencode primary assembly GRCh37	k4mLJvbFzZiw3o6SL8hh63V2u7AjDMrE	84
GRCh38-p14-46-gencode	hg38	gencode	release 46 of gencode GRCh38	tkRdBlSp5hewK4OpEJC87J9pw-ac0vOa	706
GRCh38-primary-assembly-46-gencode	hg38	gencode	release 46 of gencode primary assembly GRCh38	tmnbiAyj2fke68d_TYjq2g487US8C15r	194
GRCh37-primary-assembly-46-gencode	hg19	gencode	release 46 of gencode primary assembly GRCh37	k4mLJvbFzZiw3o6SL8hh63V2u7AjDMrE	84
hg18-ucsc	hg18	ucsc	2006 release of hg18 ucsc	H9er8ocYfIN2TOfyf6zMyeXXm7trXGP7	49
GRCh38-full-decoy-hla-ddbj	hg38	ddbj	ddbj GRCh38_full_analysis_set_plus_decoy_hla.fa	gHcfbUVnFzHv3QSqz2sSqVHdUQbDO8N5	3366
homo-sapiens-assembly38-ddbj	hg38	ddbj	ddbj Homo_sapiens_assembly38.fasta	gHcfbUVnFzHv3QSqz2sSqVHdUQbDO8N5	3366
hg38-ddbj	hg38	ddbj	ddbj hg38.fa.gz	NTeQ1GQMt2ocCFkS8Z3_qkvetZjabWSt	455
GRCh38-ena-29	hg38	ENA	ref genome hosted on ENA Genome Reference Consortium Human Build 38 patch release 14 (GRCh38.p14)	lWRRNMNypacEjnJCy-AYiDNUPy1brQGC	68
GRCh38-ena-15	hg38	ENA	ref genome hosted on ENA Genome Reference Consortium Human Build 38 (GRCh38.p0)	6chutju9QVJW0rdA-wgubHbtoTQ42o-6	67
hg38-igenomes-ucsc	hg38	igenomes	igenomes hosted hg38	jFm0Uca8a7vK2cbuIQgBopjBilgCFheD	195
hg19-igenomes-ucsc	hg19	igenomes	igenomes hosted hg19	GOIHeGSorDrbznRxihs5rIb6vTiTKaw7	25
hg18-igenomes-ucsc	hg18	igenomes	igenomes hosted hg18	ieWVCws5MC2QFRKgH9QcN3u5_Y_3hPG6	25
hg38-refgenie	hg38	refgenie	refgenie's The GCA_000001405.15 GRCh38_no_alt_analysis_set from NCBI	EiFob05aCWgVU_B_Ae0cypnQut3cxUP1	195
hg38-primary-refgenie	hg38	refgenie	refgenie's UCSC hg38 assembly with primary chromosomes only	Ba88PY52_qeifhJrgUXyin6UITdXNsg3	25
hg18-refgenie	hg18	refgenie	refgenie's NCBI36 reference sequence from UCSC. The individual chromosome files were concatenated prior to the asset building	YfZ0rklv8KY9DCtqG0iIX16zsgbuBgmM	49
hg19-refgenie	hg19	refgenie	refgenie's he GRCh37 reference sequence from UCSC	ThZcNYiLuWWL86NdJ8dvvJG15K9mW3Fo	93
hg38-broad	hg38	broad	broad institute hosted hg38	gHcfbUVnFzHv3QSqz2sSqVHdUQbDO8N5	3366
hg38-noALT-noHLA-noDecoy-broad	hg38	broad	broad institute hosted hg38-noALT-noHLA-noDecoy	EiFob05aCWgVU_B_Ae0cypnQut3cxUP1	195
b37-broad	hg19	broad	broad institute b37	eN7J_gZz_meakMCeXXBEvY_njignMPxl	85
GRCh37-igenomes-ensembl	hg19	igenomes	igenomes hosted GRCh37	lXa-sGAmSafYXHN4iEwup0EsQh5F1krA	25
GRCh38-igenomes-ncbi	hg38	igenomes	igenomes hosted GRCh38	EiFob05aCWgVU_B_Ae0cypnQut3cxUP1	195
GRCh38-igenomes-build37-2-ncbi	hg38	igenomes	igenomes hosted GRCh38 build 37.2	Dx_M8skbJqROkfXhhQRtWejcCyewRbdL	25
GRCh38-igenomes-build37-1-ncbi	hg38	igenomes	igenomes hosted GRCh38 build 37.1	wdpZbFN0pd92H2VeKZBAp3riQN4nJXkK	25
GRCh38-igenomes-build36-3-ncbi	hg38	igenomes	igenomes hosted GRCh38 build 36.3	vRjC5qM1Tc-fFjJo0TGRw4CVjFhmLG0f	25
GRCh38-igenomes-decoy-ncbi	hg38	igenomes	igenomes hosted GRCh38 decoy	FnbS0xDAGOePD7lp6Xt0vnbcASzkk3gk	2580
hs37-1kg	hg19	1000genomes	hs37-1kg genome referenced on HL blog 2017 1000 Genomes Project (1000G) reference in 2010	5SdkZCnuZL2YIptqjSBZfupo_O7HpD_B	84
hs37d5	hg19	1000genomes	hs37d5 genome referenced on HL blog 2017 hs37 with decoy (recommended GRCh37)	Q3xii3AkJDCTXSO6Vg13kjbOutQu0KP9	86
hs38	hg38	ncbi	GRCh38 no-alt analysis set (recommended GRCh38)	EiFob05aCWgVU_B_Ae0cypnQut3cxUP1	195
hs38DH	hg38	1000genomes	GRCh38 with ALT decoy and HLA genes (not recommended)	gHcfbUVnFzHv3QSqz2sSqVHdUQbDO8N5	3366
hs37d5.fa	hg19	ddbj	ddbj hs37d5.fa	Q3xii3AkJDCTXSO6Vg13kjbOutQu0KP9	86

Supplemental Table S5: *Human reference genomes used for main analysis. N=number of sequences present in the reference genome.*

bioRxiv preprint doi: https://doi.org/10.1101/2025.10.06.680641; this version posted October 6, 2025. The copyright holder for this preprint (which was not certified by peer review) is the author/funder, who has granted bioRxiv a license to display the preprint in perpetuity. It is made available under aCC-BY 4.0 International license.

Genome	Common	Authority	Description	Top-level digest	N
GRCm39-fasta-full-analysis	mm39	ncbi	GCA_000001635.9_GRCm39_full_analysis_set from NCBI	-e70JAQq4NJDg8-1Ab2XhHu6yYjeW-zu	61
GRCm39-fasta-genomic	mm39	ncbi	GCF_000001635.27_GRCm39_genomic from NCBI	fXjBOJjw-DYsSnnfDBl5vtZu1N7lbnUl	61
GRCm38.p6-fasta-genomic	mm38	ncbi	GCF_000001635.26_GRCm38.p6_genomic from NCBI	jpOqOhddb15iOm2SIdJSjsf-U5Uu7Def	239
GRCm38.p6-fasta-genomic-GCA	mm38	ncbi	GCA_000001635.8_GRCm38.p6_genomic.fna.gz from NCIBI	JL56x8L1q1Fs_-jHvZxBG01Vitac-CmO	239
GRCm39-fasta-genomic-GCA	mm39	ncbi	GCA_000001635.9_GRCm39_genomic.fna.gz from NCBI	PM8ODmBlTISp4Onv0aSBFaAfi3QVCGzx	61
GRCm39-fasta-full-analysis-ucsc-ids	mm39	ncbi	GCA_000001635.9_GRCm39_full_analysis_set.fna.gz with ucsc ids from NCBI	-e70JAQq4NJDg8-1Ab2XhHu6yYjeW-zu	61
mm10-ucsc-initial-soft-masked	mm10	ucsc	mm10 from ucsc representing the 2012 release soft masked	99TjKCwZJJLjpBqkLpTgC2E_Y3OgKtMz	66
mm10-ucsc-initial-hard-masked	mm10	ucsc	mm10 from ucsc representing the 2012 release hard masked	FTBYBUoMhkOJ_-8lWpERVTxe62kstAol	66
mm10-ucsc-p6-soft-masked	mm10	ucsc	mm10 p6 from ucsc soft masked	M7ZWnvUTT06JREJnMb_7UGwgGaG0-13s	239
mm10-ucsc-p6-hard-masked	mm10	ucsc	mm10 p6 from ucsc hard masked	9k1WrFA4Ys2fPifOOVswhOEdurvsaLfl	239
mm39-ucsc-initial-soft-masked	mm39	ucsc	mm39 initial ucsc soft masked	KPagVaXI4XwQ1D0L0EW4eEuVl-otaAtX	61
mm39-ucsc-initial-hard-masked	mm39	ucsc	mm39 initial ucsc hard masked	3MS1-4k87pZ0-C80QDoUvhFmC0usPH28	61
mm9-ucsc-initial	mm9	ucsc	mm9 initial 2007	vygX07e7feibvucSnWj6hRScGMfc7B6P	35
GRCm39-primary-113-ensembl	mm39	ensembl	ensembl 113 primary assembly	2Ls1P5eUdKbvtOhjJx3s2R5r0_I-IB5Z	61
GRCm39-primary-rm-113-ensembl	mm39	ensembl	ensembl 113 primary assembly rm	D-6wf8dsOttiVNnLSImSglRJvw_8Zr_j	61
GRCm39-toplevel-113-ensembl	mm39	ensembl	ensembl 113 top level	XVhfRj6PCzLoGiEeXjpFv7vriVN02aPc	61
GRCm39-toplevel-rm-113-ensembl	mm39	ensembl	ensembl 113 top level rm	qcT5VVX5G3mN2O9OqeFR-F0POVuY2oGw	61
GRCm39-primary-sm-113-ensembl	mm39	ensembl	ensembl 113 primary sm	2Ls1P5eUdKbvtOhjJx3s2R5r0_I-IB5Z	61
GRCm39-toplevel-sm-113-ensembl	mm39	ensembl	ensembl 113 top level sm	XVhfRj6PCzLoGiEeXjpFv7vriVN02aPc	61
GRCm39-toplevel-65-ensembl	mm9	ensembl	ensembl NCBIM37.65 top level	3rgz8-_XPSiTUYPamUTRF3DArhAhTint	211
GRCm39-toplevel-rm-65-ensembl	mm9	ensembl	ensembl NCBIM37.65 rm top level	JPyo8AqZzCyVaUx1lAkk6LbpyQPX4VUB	211
GRCm39-primary-M36-gencode	mm39	gencode	gencode primary release m36 mm39	WsOG-InFnIta0rqSy1KUBjrFrukbnE5j	61
GRCm39-all-M36-gencode	mm39	gencode	gencode all release m36 mm39	WsOG-InFnIta0rqSy1KUBjrFrukbnE5j	61
GRCm39-all-M1-gencode	mm9	gencode	gencode all M1 mm9 corresponds to Ensembl version 65	qtPKGcXii2OuiyIoDA9K0jSKR62qCyzd	211
mm10-refgenie-ncbi	mm10	refgenie	refgenie's GCA_000001635.5 seqs_for_alignment_pipelines from NCBI	wsDErYxgCXiPnb2FWZ4sXtx3B0YyruRu	66
mm10-refgenie-ucsc	mm10	refgenie	refgenie's UCSC mm10 assembly with primary chromosomes only	dMjpOU7EvpeZVb0gpPoZ7prNaxOu88Ta	22
GRCm38-igenomes-ensembl	mm38	igenomes	igenomes's GRCm38 from Ensembl	TQORtixTJqM3Su9dmtACKc7hNHAceE4I	22
NCBIM37-igenomes-ensembl	mm37	igenomes	igenomes's NCBIM37 from Ensembl	6-UTIAyR94-nanfrhd_sAF6oHLyMd0zH	22
mm37-build37.1-igenomes-ncbi	mm37	igenomes	igenomes's ncbi build 37.1 for mm37	6-UTIAyR94-nanfrhd_sAF6oHLyMd0zH	22
mm37-build37.2-igenomes-ncbi	mm37	igenomes	igenomes's ncbi build 37.2 for mm37	6-UTIAyR94-nanfrhd_sAF6oHLyMd0zH	22
mm38-igenomes-ncbi	mm38	igenomes	igenomes's NCBI GRCm38	TQORtixTJqM3Su9dmtACKc7hNHAceE4I	22
mm9-igenomes-ucsc	mm9	igenomes	igenomes's UCSC mm9	4mvptys3ckGgiUCcly4HOHB40IhpwwVT	22
mm10-igenomes-ucsc	mm10	igenomes	igenomes's UCSC mm10	hW3Ba5zoufl3-MGXQESlWXjsW56R5vPG	22
GRCm39-ena-09	mm39	ENA	ref genome hosted on ENA Genome Reference Consortium Mouse Build 39	ABEupc6KHmxtHGarfWFXTmu9mUcNnfoM	40
GRCm39-ena-03	mm38	ENA	ref genome hosted on ENA Genome Reference Consortium Mouse Build 38 patch release 1 (GRCm38.p1)	bLbjXXCz_5qAonaDXcVuadx65QZkC7mb	50
GRCm39-ena-02	mm38	ENA	ref genome hosted on ENA Genome Reference Consortium Mouse Build 38	bLbjXXCz_5qAonaDXcVuadx65QZkC7mb	50

Supplemental Table S6: *Mouse reference genomes used for main analysis. N=number of sequences present in the reference genome.*
